# Dr. Child on Indigestion

**Published:** 1847-04

**Authors:** 


Art. I.—*On Indigestion and certain Bilious Disorders often conjoined with it. To which are added, Short Notes on Diet.* By George Child, m.d., Physician to the Westminster General Dispensary.—*London*, 1847. 8vo, pp. 219.

We cannot say that we have met in this work with any novel facts, or with any masterly grouping of old ones. The book, in short, is just another of those (of which there are already a great deal too many) which fifty or a hundred practitioners of medium intelligence, industry, and observation could easily write. When we say this, we merely mean to intimate that in Dr. Child's volume we are not to look for any essentially new views, new inductions, or new plans of treatment. The work is divided into twenty chapters, of part of the contents of which we propose to give a cursory notice.

In chapters first and second, we find nothing whatever new. We think Dr. Child's definition of dyspepsia, at the very outset, may be objected to as at least incomplete. It is "habitual uneasiness while the food in the stomach is being converted into chyme." Now though, according to the strict etymology of the word dyspepsia (which, however, Dr. Child does not refer to), the above definition may pass, yet practically and as regards the word indigestion (which the author allows to be a synonyme of dyspepsia) the case is different. For undoubtedly indigestion is something *more* than mere habitual "uneasiness" in the process of chymification. It is also *imperfect* chymification, or we may say (rather, however, to illustrate our meaning, than to express ourselves with philosophic accuracy) it is *morbid* chymification. There are, indeed, cases in which chymification is obviously most imperfect, but which are characterized by little or no pain or uneasiness, at least in the stomach itself.

At p. 7 et seq. Dr. Child notices the effects of food undue in quality or quantity, as leading to indigestion, and here he sets himself to repudiate the notion that "the diet of the rich is more calculated to produce indigestion than that of the poorer classes," and to cite, in order to controvert, the everlastingly-quoted words of the Roman satirist, "innumerabiles esse morbos miraris? Coquos numera." The author then proceeds to observe that as the means of the rich enable them to purchase better articles of food than the poor, and as in the case of the latter "the same dish is often warmed up again" (p. 9), so, for these and sundry other reasons, the rich, as regards diet and cookery, are better off than the poor. Here, again, we think there is slight special pleading, or rather a misapprehension of the true spirit of the objection which the author is combating. No one ever, we believe, would contend that a good article of food is preferable to an inferior one: a sufficiently cooked to an imperfectly cooked dish. But the point aimed at in the query of the satirist and the real *practical* part of the question is, whether the plain and even coarse diet of the poor is not found, *provided only it be sufficient in quantity,* to lead *actually* to less disease, and to the formation of fewer of those artificial tastes, from which disease almost unavoidably results, than the more recondite cookery of the rich does? We think that, looking at the simple and actual results of the two "systems," the question admits but of one answer, though, perhaps, handled as it is by the author, it is one fitted rather for discussion in a popular than a professional volume.

At chapter vii, p. 83, "the various pains" which characterize indigestion are treated of. Some of these pains, singular in themselves, are very singularly named and described by the author.

Taking up separately these various pains, the author gives his theory of their respective causes, and lays down what he considers the appropriate treatment. This piecemeal method of treating the subject, this attempt to make it be believed that these "pains" are susceptible of an exact pathogenical and pathological classification, is apt to be extremely hurtful in practice. Persons who are imposed on by this dogmatic nomenclature of the author (we do not use the word dogmatic in its offensive meaning) are led to suppose that a far fuller and distincter insight can be gained into the complexities and obscurities of dyspeptic derangements, than can be practically obtained. In no part of the work, perhaps, is this endeavour to give precision to subjects actually incapable of it, more visible and more unsuccessful, than in the observations on "bilious headache," "gastric headache," and "gastrobilious headache" (pp. 114 to 129 inclusive), which affections, both from the topical proximity and nervous connexions and sympathy of the organs concerned in them, and from our still great ignorance of the physiology and pathology of these, are totally insusceptible of diagnostic distinctions so precise as those attempted to be laid down by Dr. Child.

To a great extent the therapeutical directions are distributed through the work, following the descriptions of the several forms of dyspepsia, to which those directions apply; but chapter xix, page 191, is devoted to the "general treatment of indigestion," in which, however, we do not find anything materially new or important. We cannot, however, refrain from giving one quotation from this part of the work, with a view to afford the reader an opportunity of judging of the inductive powers of the author. Often a little circumstance lets us into a knowledge of an author's capacity and mode of drawing conclusions from premises. The passage we refer to occurs at page 196, and is as follows:—"The well-known virtue of nitrate of silver, internally administered, in removing morbid irritability of the stomach, led me to expect that its external application would prove a highly important counter-irritant in dyspeptic congestion. Accordingly, I have often rubbed it on the epigastrium, but I must confess I have been disappointed with its effects." It would not be easy to write a sentence more open to criticism than the above, but the reader will discover this without our aid.

Chapter xx is occupied with directions of a purely popular character, in regard to diet, &c.

We have been struck, in this volume, with the little reference to what may be called the hygienic treatment of dyspeptic disorders, which, of all others, are most benefited by such treatment. Change of scene and place and occupation, mental distraction, relaxation, excitement, fresh air, exercise, walking, riding, jumping, racing, dancing, are little, if at all, alluded to, yet a large proportion of cases of "dyspepsia and the bilious disorders often conjoined with it" would vanish under the adoption of these means alone, along, of course, with plain and moderate diet; while, *without* these means, no variety or amount of *medicinal* treatment will much or at least radically avail. But, alas! in most of our practical works, "the trail of the serpent is over all:"—drugs—drugs—nothing but drugs; trade—trade—nothing but trade; while that which mainly appertains to the more refined and higher philosophy of our noble and majestic art, is rejected as valueless, or passed by unheeded and unknown.

